# Prevalence of self-reported diabetes risk factors and integration of diabetes screening and referral at two urban HIV care and treatment clinics in Zambia

**DOI:** 10.1371/journal.pone.0275203

**Published:** 2022-09-26

**Authors:** Joy Noel Baumgartner, Namakau Nyambe, Lavanya Vasudevan, Prisca Kasonde, Michael Welsh

**Affiliations:** 1 FHI 360, Durham, NC, United States of America; 2 FHI 360, Lusaka, Zambia; 3 Family Medicine & Community Health, and Duke Global Health Institute, Duke University, NC, United States of America; Brighton and Sussex Medical School, UNITED KINGDOM

## Abstract

People living with HIV (PLWH) on antiretroviral therapy (ART) are living longer and are at risk of HIV co-morbidities including non-communicable diseases (NCDs), particularly in low-resource settings. However, the evidence base for effectively integrating HIV and NCD care is limited. The Chronic Health Care (CHC) checklist, designed to screen for multiple NCDs including a 6-item diabetes self-report screener, was implemented at two PEPFAR-supported HIV clinics in Kabwe and Kitwe, Zambia. Study objectives were to describe the HIV care and treatment population and their self-reported diabetes-related symptoms, and to evaluate provider-initiated screening and referral post-training on the CHC checklist. This cross-sectional study enrolled 435 adults receiving combination ART services. Clinic exit interviews revealed 46% self-reported at least one potential symptom, and 6% self-reported three or more symptoms to the study team, indicating risk for diabetes and need for further diagnostic testing. In comparison, only 8% of all participants reported being appropriately screened for diabetes by their health provider, with less than 1% referred for further testing. This missed opportunity for screening and referral indicates that HIV-NCD integration efforts need more fully resourced and multi-pronged approaches in order to ensure that PLWH who are already accessing ART receive the comprehensive, holistic care they need.

## Introduction

Global calls for optimizing integrated health services for HIV care and treatment clients in low-income countries are based on both the chronic and complex health needs of people living with HIV (PLWH) as well as the recognition that integrated services support a more efficient service delivery system in poorly resourced and fragmented health care systems [1–5]. In Zambia, the adult HIV prevalence is 11.1%; with a higher prevalence among urban populations and among women [6]. PLWH who are receiving combination antiretroviral therapy (cART) are living longer and are at risk of multiple HIV co-morbidities including non-communicable diseases (NCDs) that require additional care from skilled providers [7, 8].

As PLWH on treatment age and as studies reveal metabolic complications related to HIV treatment, the need for integrated care and treatment for NCDs is paramount. Integrated services are designed so that every patient contact is exploited to yield maximum value for the client, provider and community at large. While it may not be possible for a single provider to meet all the health and social services needs of PLWH, increasingly, studies are showing that screening and referral, at a minimum, are possible. For example, globally, there have been significant contributions to the literature on the integration of sexual and reproductive health services including referral strategies; however, the evidence base on how to effectively integrate NCD and HIV care and treatment is still limited [4, 9–12].

Diabetes mellitus (DM) is one of the more common NCDs globally including among PLWH with a reported prevalence ranging from 4% to 10% among the eight highest HIV prevalence countries [13]. While a recent meta-analysis found no significant association between HIV infection or treatment and DM prevalence across Africa-based studies, co-morbidities impact optimal HIV treatment and quality of life outcomes [14]. In Zambia, a combined prevalence of impaired glucose level or diabetes was estimated at 4.0% for a general population sample and a study among cART clients found that 10% had impaired fasting glucose and 5% had diabetes [7, 15]. Given these prevalence estimates, routine diabetes screening that facilitates access to early diagnoses and timely treatment among an HIV treatment-engaged population has the potential to reduce the development of future complications.

The Zambia Prevention, Care and Treatment Partnership II (ZPCT II/Bridge) project, led by the non-profit FHI 360, and funded by USAID through the U.S. President’s Emergency Plan for AIDS Relief (PEPFAR), worked in collaboration with the Ministry of Health, the Ministry of Community Development, Mother and Child Health, provincial medical offices, and district medical offices, to strengthen and expand HIV/AIDS prevention, clinical care and treatment services in Zambia from 2009–2016. The increasing incidence of NCDs in Zambia prompted FHI 360 to develop and introduce the Chronic Health Care (CHC) checklist intervention to systematically integrate NCD screening and care within government HIV services supported by ZPCT II/Bridge [16]. The checklist focused on screening for selected chronic socio-medical conditions, including *diabetes*, gender-based violence, hypertension, tuberculosis, and Prevention with Positives priorities, and referring as needed for further management.

This study focuses on the diabetes screening component of the CHC checklist and the associated referral processes which had the aim of increasing early identification and timely treatment of diabetes for PLWH. The objectives of this study are: 1) to describe the HIV care and treatment study population and their self-reported diabetes risk factors, signs and symptoms, and 2) to evaluate the prevalence of provider-initiated screening and referral for diabetes post-training on Chronic Health Care (CHC) Checklist.

## Methods

### Intervention

The CHC Checklist asks patients the following questions under a section called “Diabetes Symptom Checklist”: do you have 1) increased frequency of urination, 2) increased thirst, 3) increased water (fluid) intake, 4) increased tendency to feel hungry, 5) increased tendency to eat a lot, and 6) worsening eyesight. CHC training indicated that if a patient endorsed even one symptom, a same-day Random Blood Sugar (RBS) test (finger-prick glucometer) was indicated.

Per study protocol and aligned with national guidelines for diabetes care, for non-pregnant clients with RBS of >11.1 and presence of symptoms, this is suggestive of diabetes which called for a same day referral to the nearest diabetes specialist (intra- or inter-facility referral). For pregnant clients with RBS >7.8, a same-day referral back to antenatal care was indicated. Providers were trained to indicate action taken on CHC form under the treatment plan, which could include: facilitated referral to specialist, diabetes education, diet regulation, nutrition counseling and/or medication.

ZPCT II originally piloted the CHC checklist in 19 project-supported health facilities across six provinces providing HIV services [16]. A process evaluation revealed a need to better ensure confirmatory testing, tracking and follow-up of referrals. For this study, two large health facilities that had previously piloted the CHC checklist were selected for a more rigorous assessment of their screening and referral services based on their use of the checklist for routine screening for all care and treatment (CTC) patients. Health facility staff were re-oriented by ZPCT II clinical care technical staff on additional details for facilitated referrals [9] and follow-up procedures for diagnostics and treatment and these sites had monthly monitoring by the ZPCT II staff for technical support including ongoing emphasis on using the CHC checklist throughout the study period. Diabetes services followed guidelines from the Zambian Ministry of Health’s Second Edition of the Integrated Technical Guidelines for Frontline Health Workers [17]. It should be noted that while the CHC checklist was supported by ZPCTII and activities were in line with government approved guidelines, the form itself, as a new intervention, was not embedded into Zambia’s smartcare electronic medical record form and was paper-based in the patient’s folder. The smartcare forms included fields for noting past history of diabetes or diabetes diagnoses but there was no recording of systematic symptom screening at the time of the study.

## Materials & methods

This was a non-experimental cross-sectional study with data collected between July 2016 and February 2017. Two health facilities with large HIV Care and Treatment (CTC) patient populations were selected for the study sites:

- Buchi Main Clinic (urban health centre level) is located in the town of Kitwe, where patients in need of diabetes related services would need to be referred to Kitwe Central Hospital (third level hospital) which was within walking distance (~2 km). A site visit prior to data collection (May 2016) indicated there were on average 40 HIV patients per day, a glucometer on site, but no test strips in order to conduct in-clinic random blood sugar tests
- Kabwe General Hospital (second level hospital) in Kabwe town where patients in need of a diabetes specialist received an inter-facility referral, a short walk to another part of the same hospital. A similar site visit prior to data collection (May 2016) indicated that the ART clinic had on average 126 clients per day and no glucometer in the ART clinic. Random blood sugar tests could be conducted at the diabetes clinic within the hospital which was open one day per week or at the general laboratory. The diabetes clinic averaged 26 clients per week, but the specialist may or may not know if their client was also receiving HIV services.

The study population was all cART clinic patients aged 18 years or older who had attended cART services for at least six months. Clients receiving cART usually attend the clinic monthly for the first three months after initiating cART and then every six months thereafter. We assumed that all clients were not pregnant. Clients of cART services who become pregnant receive their HIV-related care during their antenatal care clinic visits via prevention of mother to child treatment (PMTCT) services. All cART clients were supposed to be assessed for possible comorbid NCDs at every visit via completion of the CHC checklist. Clients attending the clinic for cART services were approached for study participation as they left the health facility (exit interviews after HIV services and any other referred service onsite) by research assistants who worked in pairs at each study site. The target sample size was 400 based on time and resource constraints.

### Measures

Participants were asked a range of questions on a structured questionnaire that took less than one hour. Questions included background and household characteristics, medical history, household food security, and whether they had heard screening questions from the CHC checklist from facility staff. Participants were also (re)administered the diabetes section of the CHC checklist and physical and metabolic measurements from their SmartCare medical record were extracted (permission was part of consent).

Responses for socio-demographic and household characteristics are listed in Table 1 with clarifications noted below. Participants were asked to estimate their monthly income across nine categories in Zambian Kwachas (Less than K50, between K50-K150, between K151-K300, between K300-K450, between K451-K600, between K601-K800, between K801-K1000, between K1001-K1200, more than K1200); however for data analysis, this variable was collapsed into dichotomous categories of ≤ K1200 and >K1200.

**Table 1:** Socio-demographic characteristics of study participants, by site.

Variable^a^	Total N = 435	Kitwe N = 231	Kabwe N = 204	P-value
	% (n)	% (n)	% (n)	
Age (mean, range)	41 (20–78)	41 (20–74)	42 (20–78)	0.11
Sex				0.83
Male	45.06 (196)	44.59 (103)	45.59 (93)	
Female	54.94 (239)	55.41 (128)	54.41 (111)	
Education				0.00
None	2.55 (11)	2.18 (5)	2.96 (6)	
Some primary	14.58 (63)	6.55 (15)	23.65 (48)	
Completed primary	18.98 (82)	15.28 (35)	23.15 (47)	
Some secondary	34.72 (150)	36.68 (84)	32.51 (66)	
Completed secondary	22.69 (98)	33.19 (76)	10.84 (22)	
More than secondary	6.48 (28)	6.11 (14)	6.90 (14)	
Marital status				0.08
Never married	11.52 (50)	7.79 (18)	15.76 (32)	
Currently married	63.13 (274)	65.80 (152)	60.10 (122)	
Separated/Divorced	10.60 (46)	11.69 (27)	9.36 (19)	
Widowed	14.75 (64)	14.72 (34)	14.78 (30)	
Number of biological children (mean, range)	3.01 (0–16)	2.94 (0–9)	3.08 (0–16)	0.48
Number of household members including self (mean, range)	5.20 (1–20)	5.19 (1–12)	5.22 (1–20)	0.92
In past 4 weeks, worried household would not have enough food				0.00
No	76.55 (333)	90.04 (208)	61.27 (125)	
Yes	23.22 (101)	9.96 (23)	38.24 (78)	
[If yes to above] How often worried about food insecurity	[n = 101]	[n = 23]	[n = 78]	0.01
Rarely (1–2 times)	13.10 (57)	7.79 (18)	19.12 (39)	
Sometimes (3–10 times)	4.83 (21)	2.16 (5)	7.84 (16)	
Often (10+ times)	5.29 (23)	0.00 (0)	11.27 (23)	
Monthly household income^b^	[N = 408]	[n = 222]	[n = 186]	0.00
≤ 1200K (Zambian Kwacha)	61.52 (251)	50.00 (111)	75.27 (140)	
>1200K (Zambian Kwacha)	38.48 (157)	50.00 (111)	24.73 (46)	

^a^ If five or less were missing for any variables (N = 430 to 435), then the %s were based on non-missing data unless otherwise noted.

^b^ Income reported in Zambian kwacha (K); 1200K~ = USD $132

For the question, have you ever been diagnosed with diabetes by a health professional, the responses were no, yes, and yes (female) only during pregnancy. Both yes categories counted as endorsing this question. For “Individuals at high risk for diabetes,” a binary variable was created such that individuals with ≥3 symptoms were considered to be at high risk for diabetes. This variable was generated as follows: first, the values of the self-reported diabetes symptoms (0/1) were added up to generate a diabetes symptom score. This score had a minimum value of 0 (no symptoms) and a maximum value of 6 (all 6 symptoms present); scores 0–2 were assigned to the category <3 symptoms and scores 3–6 were assigned to the category ≥3 symptoms. For the variable “random blood sugar in chart,” RBS results were noted in mmol/l or mg/dl and for data analysis, the number of individuals for whom a RBS reading was available in their medical chart (count) is reported.

Body Mass Index (BMI) had four categories (<18.5, 18.5–24.9, 25–29.9, and 30 or more). Patient height was recorded in centimeters and weight was recorded in Kgs. BMI categories were generated as follows: (1) A new variable height in meters was created to convert the height in centimeters to meters. This conversion was achieved by dividing the height in centimeters by 100. (2) BMI scores were calculated for each respondent for whom both height and weight measurements were available using the formula weight (in Kg) / height (in m)^2^. (3) BMI scores were pooled into the aforementioned categories, which are based on CDC guidelines (ref: https://www.cdc.gov/healthyweight/assessing/bmi/adult_bmi/index.html).

### Data management & analyses

A paper-based, pre-tested questionnaire was administered by research assistants in Bemba or English depending on participant preference. Data were double entered into a Microsoft Access database. Statistical analyses were conducted using Stata v15. Tests of significance were conducted using t-tests for continuous variables and chi-square tests for binary, ordinal and categorical variables.

### Ethical considerations

Ethical approvals were received from the University of Zambia Biomedical Research Ethics Committee (UNZAREC) IRB in Lusaka, Zambia and FHI 360 in Durham, North Carolina, USA. In addition, this study received permissions from the Ministry of Health (MoH) in Zambia and the provincial authorities (Copperbelt Province and Central Province) in charge of ART facilities. Potential participants were approached for study participation upon exiting clinical services and written informed consent was obtained from all participants. If during administration of the cross-sectional patient survey, research assistants noted that a participant confirmed three or more symptoms of diabetes from the CHC checklist but said they did not get referral during their clinic visit, the study team referred them back to the clinic for follow-up care.

## Results

A total of 435 adults participated in this study, with 231 enrolled from the Kitwe health facility and 204 enrolled from the Kabwe health facility. Table 1 summarizes key socio-demographic characteristics of the participants. There were a few statistically significant differences observed between participants from the two health facilities. Compared to participants from Kabwe, a significantly higher proportion of participants from Kitwe completed secondary education, were currently married, or had higher estimated monthly household income (>K1200). Significantly fewer participants from the Kitwe health facility (9.96%, n = 23) reported worrying that their household would not have enough food in the four weeks prior to the survey compared to their counterparts from Kabwe (36.76%, n = 75).

Table 2 summarizes diabetes-related potential risk factors for the study population. Approximately 20% of the participants had a family history of diabetes and 30% met BMI cut-offs for overweight or obese. Among all participants, 54.3% self-reported no symptoms of diabetes, 45.7% self-reported at least one potential symptom, and 6.01% (n = 26) self-reported three or more symptoms. Of the 26 participants, none had been previously been told by a health provider that they had diabetes and 34.6% (n = 9) remembered the providers asking them one or more screener questions.

**Table 2:** Participant characteristics, symptoms, and risk factors related to diabetes, by site.

Variable^a^	Total N = 435	Kitwe N = 231	Kabwe N = 204	P-value
	% (n)	% (n)	% (n)	
Has family history of diabetes	19.81 (85)	15.65 (36)	24.62 (49)	0.02
Self-report of potential diabetes symptoms				
1) Increased frequency of urination	10.88 (47)	8.23 (19)	13.93 (28)	0.06
2) Increased thirst	12.50 (54)	8.23 (19)	17.41 (35)	0.00
3) Increased water intake	10.19 (44)	4.33 (10)	16.92 (34)	0.00
4) Increased tendency to feel hungry	12.53 (54)	6.09 (14)	19.90 (40)	0.00
5) Increased tendency to eat	12.06 (52)	9.13 (21)	15.42 (31)	0.05
6) Worsening sight	20.14 (87)	12.12 (28)	29.35 (59)	0.00
Endorsed 3+ potential symptoms indicating risk of diabetes	6.03 (26)	2.17 (5)	10.45 (21)	0.00
Any Random Blood Sugar results documented in HIV Care & Treatment medical chart	1.15 (5)	2.16 (5)	0.00 (0)	n/a
	**[N = 428] **	**[N = 231] **	**[N = 197]**	** **
Height [cm], (mean, range)	164 (59–187)	164 (143–182)	164 (59–187)	0.88
Weight [kg], (mean, range)	62.85 (32–118)	63.14 (32–108)	62.52 (34–118)	0.61
Body Mass Index				
<18.5 (Underweight)	10.75 (46)	6.93 (16)	15.23 (30)	0.01
18.5–24.9 (Normal)	59.11 (253)	61.47 (142)	56.35 (111)	0.28
25–29.9 (Overweight)	19.86 (85)	21.65 (50)	17.77 (35)	0.32
≥30 (Obese)	10.28 (44)	9.96 (23)	10.66 (21)	0.81
Ever diagnosed with diabetes by health professional	2.09 (9)	0.44 (1)	3.98 (8)	0.01

^a^ If five or less were missing for any variables (N = 430 to 435), then the %s were based on non-missing data unless otherwise noted.

Of the 435 participants in the study, only 2.16% (n = 5) participants from Kitwe had a recent non-zero random blood sugar (RBS) reading recorded in their medical chart. No participants from Kabwe had a recent random blood sugar reading recorded in their medical chart. Of those five participants with RBS data, all had self-reported experiencing 3+ potential diabetes symptoms; however, only one reported that a provider had asked them about these symptoms.

Compared to participants from Kitwe, more participants from Kabwe reported having a family history of diabetes or being diagnosed by a health provider as having diabetes. Correspondingly, a significant number of participants from Kabwe self-reported three or more symptoms associated with diabetes and hence, were classified as being at high risk for the disease. While no significant differences were observed in the mean height or weight of participants from the two health facilities, participants from Kabwe were more likely to be underweight (BMI<18.5).

**Fig 1:**
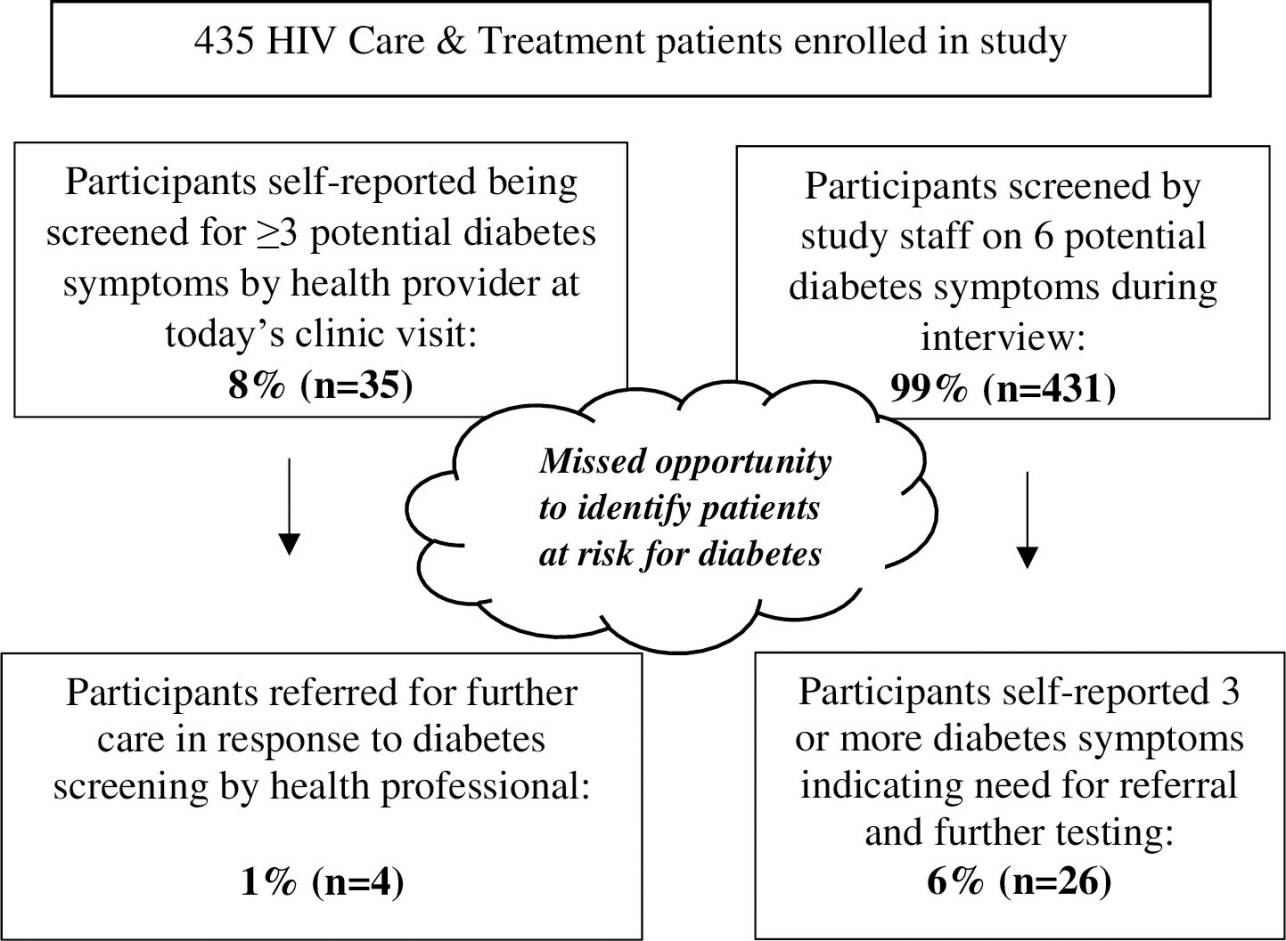
Integration of diabetes screening and referral within HIV care and treatment services.

Fig 1 shows the integration of diabetes screening and referral in the continuum of care. The study team screened 99% (n = 431) of the study population for self-reported diabetes symptoms, with 6% (n = 26) classified as being at high risk for diabetes. In comparison, only 8% (n = 35) of the participants reported being screened for diabetes symptoms with three or more questions by their health provider, with less than 1% (n = 4) being referred for further care. All four participants who had been referred by their HIV provider indicated that they had followed up on the referral.

Exploratory descriptive analyses of patient characteristics associated with self-endorsing 3+ symptoms during the survey (n = 26) revealed that household food insecurity and family history of diabetes may be more likely to be reported among those with 3+ symptoms (S1 Table; formal statistics not conducted due to sample size). Exploratory descriptive analyses of patient characteristics associated with being asked screening questions by the provider (n = 35) did not reveal any noticeable differences (S2 Table; formal statistics not conducted due to sample size)

## Discussion

People living with HIV in low-resource settings are in need of integrated HIV care and treatment services in order to meet their comprehensive health needs. While HIV care and treatment services have been increasingly successful in reducing HIV-related morbidity and mortality, screening for and provision of non-HIV-related health services for PLWH has been slower to scale [11, 12, 18]. This study revealed insufficient routine screening for diabetes at two large HIV care and treatment clinics in urban towns in Zambia. It also documents the prevalence of self-reported endorsement of possible diabetes symptoms which could be helpful for future community-based screening programs.

Our study indicates that 6% of cART clients in our study sites reported experiencing three or more possible symptoms of diabetes that would warrant immediate follow-up diagnostics and potential treatment plans for diabetes. In addition, a sizable number of cART clients were also reporting family histories of diabetes and/or their medical charts indicated BMIs above the normal category which are also risk factors for developing diabetes. The CHC Checklist and associated training actually indicated that any clients who endorsed one or more symptoms may need a referral or at least follow-up questioning to ascertain the root of cause of the symptoms. The gap identified in routine screening was much larger than our team anticipated. The study team had originally focused survey questions on barriers to following up on referrals, not realizing how few clients would even be fully screened. We acknowledge that even under ideal implementation scenarios, the CHC checklist (and random blood glucose tests) will not detect every potential case of diabetes, but endorsing 3+ symptoms would be concerning enough to warrant further clinical investigation. That said, our study did demonstrate a lack of infrastructure capacity in an already overstretched health system and perhaps some provider misunderstandings regarding the importance of an integrated diabetes program given their previous training on the checklist.

One potential reason for the low screening rates could be that prior to study initiation, site visits with the providers revealed challenges that facilities faced obtaining both glucometers and the test strips. Even when the supplies were directly provided to the HIV clinics during the study period, the supplies were frequently removed and placed in the facility’s main laboratory, available to any facility patient and quickly running out. These diabetes related supply issues have been documented in similar HIV clinic settings [19]. Given this logistics situation, it could be that if the HIV providers knew there would be an eventual diagnostic barrier, they preemptively did not screen.

Alternatively, the original barriers to using the CHC checklist during the pilot phase may have not been fully resolved with the ongoing technical support from FHI 360 staff via the USAID-funded ZPCT II program—namely, that providers indicated they were already too busy with high patient loads and the CHC checklist was not yet permanently embedded in the Ministry of Health Smartcard patient charts. Although ZPCT II staff worked in close coordination with the Ministry of Health and provincial health offices, the checklist could have still been de-prioritized by individual providers who did not recognize it as valuable routine data and patient care. Other projects have documented that local ownership is key and lack thereof may lead to clinic resistance without additional compensation or incentives for new clinical procedures [20].

This study had a few limitations. Data were only collected from two health facilities from two urban sites and therefore may not be generalizable to the general population of PLWH in Zambia, particularly rural populations. In addition, while we attempted to recruit every eligible outpatient at these two clinics, we may have missed some who may have been more or less vulnerable to co-morbidities such as diabetes. We also note that we did not conduct confirmatory diagnostic testing for diabetes for those with 3+ symptoms as this was implementation research; thus we do not know the true prevalence of diabetes among our study sample. Finally, we do not have a comparison group from facilities that were not linked to ZPCT and their technical support including the CHC checklist. Thus, the estimates of diabetes risk could be higher or lower at other ART clinics in Zambia.

The growing demand for care for PLWH that have co-morbidities in Southern Africa shows the need for more innovative ways to integrate management of communicable and non-communicable diseases. HIV care and treatment clinics need to proactively manage NCDs like diabetes for their patients in order reduce their risk for morbidity and mortality. However, if HIV providers do not fully appreciate the need to routinely screen all PLWH for NCDs, then they are less likely to adhere to screening protocols. Likewise, stockouts for diabetes diagnostic equipment is a further disincentive for screening. On a positive note, at our sites, the diabetes specialists at both of the referral hospital clinics were ready and willing to serve more clients living with HIV, they just wanted more training so they could better serve those clients with a deeper understanding of their HIV management plans. Upon reflection and in line with other study findings, we realize that a dedicated champion for HIV-NCD integration sitting in the HIV clinic (e.g. manager or senior clinician) may have ensured better integrated care amidst these daily challenges [21]. There is evidence that champions can help integrate a new innovation into a complex organizational context via their positions of influence and sustained engagement [22]. Diabetes care and treatment in low-resource contexts is already challenging [23]. Going forward, HIV-NCD integration efforts will need a more fully resourced and multi-pronged infrastructure approach that includes training, supervision, a commitment to eliminating stockouts, and screening/referral data that is fully embedded and required in patient charts to ensure that PLWH get the comprehensive, holistic care they need.

## Supporting information

S1 Table Characteristics of participants stratified by their self-reported diabetes symptom score (<3 items versus 3+ items). (DOCX)

S2 Table Characteristics of participants stratified by whether they stated that provider screened them for potential diabetes symptoms (asked about <3 diabetes symptoms versus asked about 3+ diabetes symptoms). (DOCX)

S1 Questionnaire (DOCX)
