# Supplementary material for: Prevalence of self-reported diabetes risk factors and integration of diabetes screening and referral at two urban HIV care and treatment clinics in Zambia
Source: PLoS One. 2022 Sep 26;17(9):e0275203. doi: 10.1371/journal.pone.0275203 (PMC9512175; doi:10.1371/journal.pone.0275203)
Supplement: S1 Table — (DOCX) [file pone.0275203.s001.docx]

| **S1 Table: Characteristics of participants stratified by their self-reported diabetes symptom score (<3 items versus 3+ items)*** | | | | |
| --- | --- | --- | --- | --- |
|  | **Self-reported diabetes symptoms** | | | |
|  | **Less than 3** | | **3 or more** | |
| **Age, mean (SD)** | 41.30 | (10.32) | 41.50 | (8.31) |
| **Sex, n (%)** |  |  |  |  |
| Male | 183 | 45.19% | 11 | 42.31% |
| Female | 222 | 54.81% | 15 | 57.69% |
| **Education, n (%)** |  |  |  |  |
| None | 10 | 2.49% | 0 | 0.00% |
| Some primary | 58 | 14.43% | 5 | 19.23% |
| Completed primary | 74 | 18.41% | 8 | 30.77% |
| Some secondary | 145 | 36.07% | 3 | 11.54% |
| Completed secondary | 90 | 22.39% | 8 | 30.77% |
| More than secondary | 25 | 6.22% | 2 | 7.69% |
| **Marital Status, n (%)** |  |  |  |  |
| Never married | 43 | 10.64% | 6 | 23.08% |
| Currently married | 257 | 63.61% | 15 | 57.69% |
| Separated/Divorced | 42 | 10.40% | 4 | 15.38% |
| Widowed | 62 | 15.35% | 1 | 3.85% |
| **Number of biological children, mean (SD)** | 3.02 | (2.01) | 2.77 | (2.69) |
| **Number of household members, mean (SD)** | 5.15 | (2.45) | 6.23 | (2.87) |
| **Monthly household income, mean (SD)** |  |  |  |  |
| ≤K1200 | 234 | 61.42% | 16 | 66.67% |
| >K1200 | 147 | 38.58% | 8 | 33.33% |
| **In the past 4 weeks, worried household would not have enough food, n (%)** |  |  |  |  |
| No | 320 | 79.01% | 10 | 38.46% |
| Yes (if yes, answers question below) | 85 | 20.99% | 16 | 61.54% |
| **How often worried about food insecurity, n (%)** |  |  |  |  |
| Rarely (1-2 times) | 47 | 55.29% | 10 | 62.50% |
| Sometimes (3-10 times) | 18 | 21.18% | 3 | 18.75% |
| Often (10+ times) | 20 | 23.53% | 3 | 18.75% |
| **BMI, n (%)** |  |  |  |  |
| <18.5 | 41 | 10.25% | 5 | 20.00% |
| >=18.5 & <25 | 240 | 60.00% | 12 | 48.00% |
| >=25 & <30 | 80 | 20.00% | 4 | 16.00% |
| >=30 | 39 | 9.75% | 4 | 16.00% |
| **Family history of diabetes, n (%)** |  |  |  |  |
| No | 327 | 81.75% | 14 | 53.85% |
| Yes | 73 | 18.25% | 12 | 46.15% |

*only non-missing data reported
