# Supplementary material for: Prevalence of self-reported diabetes risk factors and integration of diabetes screening and referral at two urban HIV care and treatment clinics in Zambia
Source: PLoS One. 2022 Sep 26;17(9):e0275203. doi: 10.1371/journal.pone.0275203 (PMC9512175; doi:10.1371/journal.pone.0275203)
Supplement: S2 Table — (DOCX) [file pone.0275203.s002.docx]

| **S2 Table: Characteristics of participants stratified by whether they stated that provider screened them for potential diabetes symptoms (asked about <3 diabetes symptoms versus asked about 3+ diabetes symptoms)*** | | | | |
| --- | --- | --- | --- | --- |
|  | **Provider screened diabetes symptoms** | | | |
|  | **Less than 3** | | **3 or more** | |
| **Age, mean (SD)** | 41.43 | (10.09) | 39.34 | (11.12) |
| **Sex, n (%)** |  |  |  |  |
| Male | 180 | 45.57% | 16 | 45.71% |
| Female | 215 | 54.43% | 19 | 54.29% |
| **Education, n (%)** |  |  |  |  |
| None | 9 | 2.30% | 1 | 2.86% |
| Some primary | 55 | 14.03% | 6 | 17.14% |
| Completed primary | 74 | 18.88% | 7 | 20.00% |
| Some secondary | 139 | 35.46% | 10 | 28.57% |
| Completed secondary | 89 | 22.70% | 9 | 25.71% |
| More than secondary | 26 | 6.63% | 2 | 5.71% |
| **Marital Status, n (%)** |  |  |  |  |
| Never married | 48 | 12.18% | 2 | 5.71% |
| Currently married | 248 | 62.94% | 24 | 68.57% |
| Separated/Divorced | 42 | 10.66% | 4 | 11.43% |
| Widowed | 56 | 14.21% | 5 | 14.29% |
| **Number of biological children, mean (SD)** | 3.00 | (2.07) | 2.77 | (1.88) |
| **Number of household members, mean (SD)** | 5.16 | (2.47) | 5.59 | (2.69) |
| **Monthly household income, mean (SD)** |  |  |  |  |
| ≤K1200 | 227 | 60.70% | 21 | 67.74% |
| >K1200 | 147 | 39.30% | 10 | 32.26% |
| **In the past 4 weeks, worried household would not have enough food, n (%)** |  |  |  |  |
| No | 308 | 77.97% | 23 | 65.71% |
| Yes (if yes, answers question below) | 87 | 22.03% | 12 | 34.29% |
| **How often worried about food insecurity, n (%)** |  |  |  |  |
| Rarely (1-2 times) | 50 | 57.47% | 5 | 41.67% |
| Sometimes (3-10 times) | 17 | 19.54% | 4 | 33.33% |
| Often (10+ times) | 20 | 22.99% | 3 | 25.00% |
| **BMI, n (%)** |  |  |  |  |
| <18.5 | 42 | 10.77% | 4 | 11.43% |
| >=18.5 & <25 | 230 | 58.97% | 23 | 65.71% |
| >=25 & <30 | 79 | 20.26% | 5 | 14.29% |
| >=30 | 39 | 10.00% | 3 | 8.57% |
| **Family history of diabetes, n (%)** |  |  |  |  |
| No | 313 | 80.05% | 30 | 88.24% |
| Yes | 78 | 19.95% | 4 | 11.76% |

*only non-missing data reported
